# Magnetic Resonance Colonography for Fibrosis Assessment in Rats with Chronic Colitis

**DOI:** 10.1371/journal.pone.0100921

**Published:** 2014-07-07

**Authors:** Chloé Melchior, Emilien Loeuillard, Rachel Marion-Letellier, Lionel Nicol, Paul Mulder, Charlène Guerin, Christine Bôle-Feysot, Moutaz Aziz, Pierre Déchelotte, Pierre Vera, Guillaume Savoye, Céline Savoye-Collet

**Affiliations:** 1 INSERM UMR 1073, Institute for Biomedical Research, Rouen University, Rouen, France; 2 Gastroenterology Department, Rouen University Hospital, Rouen, France; 3 INSERM U644, Institute for Biomedical Research, Rouen University, Rouen, France; 4 Pathology Department, Rouen University Hospital, Rouen, France; 5 Nutrition Department, Rouen University Hospital, Rouen, France; 6 Radiology Department, Rouen University Hospital, Rouen, France; 7 QUANTIF-LITIS EA 4108, Rouen University, Rouen, France; Duke University Medical Center, United States of America

## Abstract

**Background:**

Magnetic resonance colonography (MRC) has been developed to assess inflammatory bowel diseases. We aimed to assess the feasibility of MRC in rats with TNBS-induced chronic colitis and to confront imaging results with fibrosis and stenosing features of the model.

**Materials and Methods:**

Chronic colitis was induced in 12 rats by weekly intra-rectal injection of increasing doses of TNBS for 6 weeks, while 8 control rats received the vehicle. At week 7, MRC was performed. Fibrosis scores were assessed and fibrosis mediators measured.

**Results:**

Chronic colitis was associated with significant body weight loss (p<0.0001) and higher colon weight/length compared to controls (p = 0.0004). Fibrosis mediators and histological scores were significantly higher in rats with TNBS than in controls: α-SMA expression (0.9 versus 0.61, p = 0.0311) and fibrosis score (p = 0.0308). Colon wall thickness was higher in rats with TNBS than in controls: maximal thickness (2.38 versus 0.74 mm, p<0.0001) and minimal thickness (1.33 versus 0.48 mm, p<0.0001). Wall signal intensity on T2w images was higher in rats with TNBS than in controls (9040 versus 6192, p = 0.0101) and correlated with fibrosis score (r = 0.5214; p = 0.04). Luminal narrowing was higher in rats with TNBS (50.08 versus 10.33%, p<0.0001) and correlated with α-SMA expression (r = 0.5618; p = 0.01). Stenosis was observed in 7/9 rats with TNBS and in no controls (p = 0.0053).

**Conclusions:**

MRC is feasible and easily distinguishes rats with colitis from controls. MRC signs correlated with fibrosis parameters. MRC evaluation may be part of a new anti-fibrosis drug assessment in experimental models of chronic colitis.

## Introduction

Inflammatory Bowel Diseases (IBD) are characterized by recurrent clinical flares contrasting with periods of remission. However, lesions may not follow this pattern and are probably more progressive [1]. The intestinal damages associated with this continuous progression are the basis of the changing phenotype of the disease overtime [2], [3]. These changes, which may be either penetrating or fibrosing, affect two thirds of patients at 20 years and lead most of them to surgery at least once during their life [4]. Among stenosing lesions, fibrosis is usually refractory to anti-inflammatory drugs [5], [6]. Two therapeutic options may be developed: (i) earlier introduction of anti-inflammatory agents to avoid irreversible bowel damage (ii) direct targeting of fibrosing processes. Therefore, better understanding of fibrosing processes is crucial for development of new antifibrosis therapies. Faced with these needs, animal models have emerged.

Intestinal fibrosis is defined as excessive deposition of extracellular matrix. This deposition could result from chronic inflammation or deregulation of the healing process [7]. Mesenchymal cells usually produce extracellular matrix under the influence of TGF-β [8]. Epithelial and endothelial cells could be activated by TGF-β and IL-1 β to produce extracellular matrix [9].

TNBS (2,4,6-trinitrobenzene sulfonic acid) -induced colitis is a widespread model of experimental intestinal inflammation with transmural, segmental lesions and TH1 profile close to Crohn's Disease (CD) [10]. More recently, experimental IBD models with intestinal fibrosis have been developed. TNBS-induced chronic fibrosis is one such rat model, validated for chronic lesions [11].

In clinical practice, IBD and especially CD are commonly evaluated by endoscopy for mucosal assessment and by magnetic resonance (MR) for transmural lesions [12], [13] Numerous studies both in humans and animals have correlated MR images with severity of endoscopic lesions [12], [14], [15], [16], [17]. Whereas MR evaluation of inflammation is easy and becoming routine practice, assessment of fibrosing lesions is more challenging [13]. In a previous study in acute TNBS-induced lesions [16], MRC was relevant for proper assessment of acute inflammation. To our knowledge, only few data based on MR technology are available in fibrosis assessment [18], [19].

The aims of the present study were to assess the feasibility of MRC in rats with TNBS-induced chronic colitis and to confront imaging results with fibrosis and stenosing features of the model.

## Materials and Methods

### Ethics Statement

Animal care and experimentation complied with both French and European Community regulations (Official Journal of the European Community L358, 18/12/1986). Furthermore, Rachel Marion-Letellier is authorized by the French government to use the present rat model (Authorization no. 76-106). This study was approved by our Institutional Animal Care and Use Committee (Comité d'Ethique NOrmande en Matière d'EXpérimentation Animale, CENOMEXA 1112-03). Colitis induction was performed under Ketamine and Chlorpomazine anesthesia, and all efforts were made to minimize suffering.

### Rats and study design

Sprague-Dawley male rats weighing 100–150 g were obtained from Janvier (Le Genest St Isle, France). Rats were acclimated in standard cage for 7 days, with light-dark cycles of 12 hours/12 hours. They were then randomized into two groups: TNBS group (12 rats) and control group (8 rats). Water and food were provided *ad libitum*.

### Induction of chronic colitis

Chronic intestinal fibrosis was induced by intrarectal injections of TNBS (Sigma-Aldrich, Saint-Quentin Fallavier, France) weekly over six weeks (6). After 24-hour food deprivation, rats were briefly anesthetized with an intraperitoneal injection of Ketamine at 8 mg. kg^−1^ (Panpharma) and Chlorpomazine at 1 mg. kg^−1^ (Sanofi-Aventis). The TNBS group received enemas consisting of TNBS-50% ethanol in a total volume of 250 µL, with escalating weekly TNBS doses of 15, 30, 45, 60, 60, 60 mg TNBS; whereas the control group received the vehicle (250 µL of 50% ethanol). All enemas were administered by placing sedated rats in head-down position, inserting a canula into the rectum and slowly instilling the solution or the vehicle. The enema canula remained in place 60 seconds to ensure adequate delivery to the distal colon. After canula withdrawal, the rats were maintained in position for over four minutes to prevent leakage of the intracolon instillate.

### MRC procedure

Seven days after the last intrarectal injection, rats were analyzed by MR Colonography (MRC). The rats were weighed and anesthetized with an intraperitoneal injection of Thiopental (90 mg. kg^−1^). Then, they were transferred onto a cradle and placed in prone position during the procedure. Acquisition was performed with a very high magnetic field system dedicated to small animals (Bruker BioSpec 47/40USR , 4.7 Tesla). We used a T2-weighted (T2w) Rapid Acquisition with Relaxation Enhancement (RARE) sequence with and without fat suppression; TR 4630 ms, TE 30 ms, matrix 320, slice 1 mm, NEX 3, flip angle 180°, field of view (FOV) 6 cm, imaging time: 7 minutes; and a T1w Fast Slow Angle Shot (FLASH) sequence; TR 215 ms, TE 3.6 ms, matrix 256, slice 1 mm, NEX 1, flip angle 80°, FOV 6 cm; imaging time: 14 minutes. Integrated correction of respiratory movements was performed with the Intragate technique.

### MRC analysis and definition of MR semiology

MR images were analyzed with Bruker ParaVision 5.0 MRI software, specially dedicated to Bruker BioSpec 4.7 Tesla preclinical MRI. This is a software package for MRI data reconstruction, analysis and visualization. This software is dedicated to DICOM (Digital Imaging and Communications in Medicine) image file formats. It permits navigation and visualization with 2D and 3D viewers and basic functionality such as magnification, ROI (region of interest) analysis and geometrical measurement. ParaVision 5.0 software analyzed MR images. A radiologist (Céline Savoye-Collet) blind to treatment allocation interpreted images. Image quality was assessed by wall and motion artifacts each rated 0 to 3. Quality was considered as excellent without respiratory movement (0/3), good with few respiratory movements responsible for wall artifacts (1/3), and poor in other situations (2–3/3).

Measurement and signal intensity in ROI were performed in the descending part of the colon in axial plane, which anatomically allowed a cross section of the bowel. The size of ROI was adapted to the wall thickness of the rat. It was usually 0.002 cm^2^. The results presented are the mean value of measurements performed. Wall signal intensity in ROI was measured on T2w and T1w. Minimal and maximal colon wall thicknesses were measured. Luminal narrowing was calculated [(largest diameter- smallest diameter)*100/largest diameter] and expressed as percentage. Stenosis was defined as narrowing of colon lumen associated with upstream dilatation. Tubular aspect was defined as loss of colonic haustra with tubular shape of the lumen. Mucosal detachment was defined as presence of mucosal alteration with a flap in the colon lumen.

### Colon preparation

Rats were sacrificed after MRC examination at week 7. Each colon was removed and cleaned with phosphate-buffered saline (PBS) to remove fecal residues. Then, the colon was measured, weighed and photographed. Colon weight/length ratio was determined (inflammatory marker). Colon samples were stored at −80°C and one sample was fixed in formalin for histological assessment.

### Histological assessment of colon fibrosis

After formalin fixation (40%), colon samples were embedded in paraffin wax blocks and 5 µm sections were stained with hematoxylin-eosin-safran. A pathologist (Moutaz Aziz) blind to treatment allocation scored these 5 µm sections. Two semi-quantitative scores were assessed: a fibrosis score ranging from 0 (no fibrosis) to 3 (severe fibrosis) and an inflammatory score ranging from 0 (no inflammation) to 3 (severe inflammation with ulceration) were performed according to a previously validated scoring system [20]. Pictures were taken with Leica QWin software (Leica Microsystems, Bensheim, Germany).

### Colon concentration of TGF-β

Frozen colon samples were homogenized in PBS with 0.1% protease inhibitor cocktail (Sigma) and phosphatase inhibitor cocktail (Sigma). Homogenates were centrifuged (12 000 g, 15 min, 4°C) and supernatants collected. Protein concentration was determined following Bradford's colorimetric method. Samples were then analyzed with a kit (MB100B) and DuoSet ELISA Development System (R&D System, United Kingdom). Concentrations were determined by spectrophotometry at wavelength 450 nm with spectrophotometer (Metertech, Σ960, Taiwan).

### Colon expression of alpha Smooth Muscle Actin (α-SMA), p-Smad2/3 and cyclooxygenase-2 (COX-2) by Western Blot

Frozen colon samples were homogenized in PBS with 0.1% protease inhibitor cocktail (Sigma) and phosphatase inhibitor cocktail (Sigma). Homogenates were centrifuged (12 000 g, 15 min, 4°C) and supernatants were collected and stored at −80°C. Protein concentration was determined following Bradford's colorimetric method. Aliquots of supernatants containing equal amounts of protein (25 µg) were separated on 4–12% NuPAGE gel (Invitrogen) and then transferred electrophoretically to a nitrocellulose membrane (Hybond, GE Healthcare, UK). After blocking in 5% nonfat dry milk, membranes were incubated with specific primary antibodies. Primary antibodies were obtained from Epitomics (Burlingame, CA, USA) for α-SMA (1184-1), from Santa Cruz biotechnology (Tebu, Le Perray-en-Yvelines, France) for p-Smad2/3 (sc-11769), from Abcam (Cambridge, UK) for COX-2 (sc-1747) and from Sigma for β-actin. They were incubated at a dilution of 1/1000 (p-Smad2/3), 1/2000 (α-SMA), 1/250 (COX-2,) and 1/5000 (β-actin). After three washes, filter was then incubated with secondary horseradish peroxidase linked anti-rabbit IgG for p-Smad2/3, α-SMA antibody, anti-goat IgG for COX-2 and anti-mouse IgG for β-actin. To check equal loading, the blots were analyzed for β-actin expression.

### Colon Vimentin and Insulin-like Growth Factor (IGF-1) mRNA

Colon samples were frozen in liquid nitrogen and stored at −80°C before RNA preparation. Total RNA was isolated using guanidium isothiocyanate method and reverse transcribed into cDNA. mRNA expression of Vimentin, IGF-1 and internal control GAPDH was measured by quantitative RT-PCR in presence of 5 µL of Sybrgreen mix and 0.5 µL of amorce sens and antisens. The amorces were provided by Life Technologies (Saint Aubin, France). IGF-1 was performed at 62°C with F primer (CAGTTCGTGTGTGGACCAAG) and R primer (TCAGCGGAGCACAGTACATC). Vimentin was performed at 55°C with F primer (ATGAAAGTGTGGCTGCCAAGAAC) and R primer (GTGACTGCACCTGTCTCCGGTA). GAPDH control was performed at 65°C with F primer (CATCACTGCCACTCAGAAGA) and R primer (AAGTCACAGGAGACAACCG). PCR was performed with CFX95 Real Time system (Bio-Rad, Marnes-la-Coquette, France).

### Determination of Cytokine Production

Colon concentrations of interleukin-1β (IL-1 β), IL-13 and tumor necrosis factor alpha (TNF-α) were measured in duplicate by multiplex assay (R&D Systems, Abington, UK). This assay relied on use of polystyrene beads, each with a unique signature mix of fluorescent dyes that couldbe discriminated by a laser-based detection instrument (Bioplex 2200). Each bead type in the multiplex assay was coated with a specific antibody pair so as not to crossreact with other analytes in the panel.

### Statistical Analysis

Statistical comparisons were performed using GraphPad Prism 5 software. Data are expressed as mean ± SD and number (percentage). Gaussian distribution was checked with Kologorov-Smirnov test. Parametric quantitative data were analyzed with Student t-test and non-parametric quantitative data with Mann Whitney test. Qualitative data were analyzed with chi-square test and Fisher's test for low number (n<5). Correlation between parametric quantitative data was analyzed by simple linear regression and Pearson's correlation. For correlation between non-parametric data, Spearman rank-order correlation was used and Spearman correlation coefficient r was calculated. A p value equal to or less than 0.05 was considered significant.

## Results

Body weight was recorded on day of MRC procedure and was significantly lower in rats with TNBS than in the control group (345±57 g versus 465±9 g, p<0.0001). Colon weight/length ratio was significantly higher in the TNBS group than in the control group (29.2±9.5 g.m^−1^ versus 11.0±1.4 g.m^−1^, p = 0.0004).

Histological score of fibrosis was higher in the TNBS than in the control group (p = 0.031). Mild, moderate or severe fibrosis was observed on histological score in 6 out of 9 rats in the TNBS group, whereas only 1 control rat had signs of histological fibrosis.

Regarding fibrosis markers, there were significant differences between the TNBS and the control group. Colon production of α-SMA was higher in the TNBS than in the control group (0.90±0.25 versus 0.61±0.19, p = 0.0311). Colon production of IL-13 was higher in the TNBS than in the control group (3.70±4.19 pg.mg^−1^ versus 0.31±0.18 pg.mg^−1^, p = 0.05), likewise for colon production of p-SMAD2/3 (1.17±1.69 versus 0.31±0.097, p = 0.05). Colon production of TGF-β was no different between the two groups (27±5 pg.mg^−1^ versus 25±7 pg.mg^−1^, p = 1.0) likewise RNA levels of vimentin (p = 0.3573) and IGF-1 (p = 0.3969).

Inflammation was also present on histological specimens in all rats with TNBS, whereas only 1 control rat had signs of histological inflammation (p = 0.0016). Inflammatory parameters were significantly higher in the TNBS than in the control group for colon production of COX-2 (0.79±0.44 versus 0.21±0.10, p = 0.0046). Colon production of IL-1β was no different between the two groups (224±231 pg.mg^−1^ versus 94±26 pg.mg^−1^, p = 0.0728) likewise TNF-α production (1.68±1.29 pg.mg^−1^ versus 1.87±0.69 pg.mg^−1^, p = 0.4079).

### MRI data

**Figure 1:**
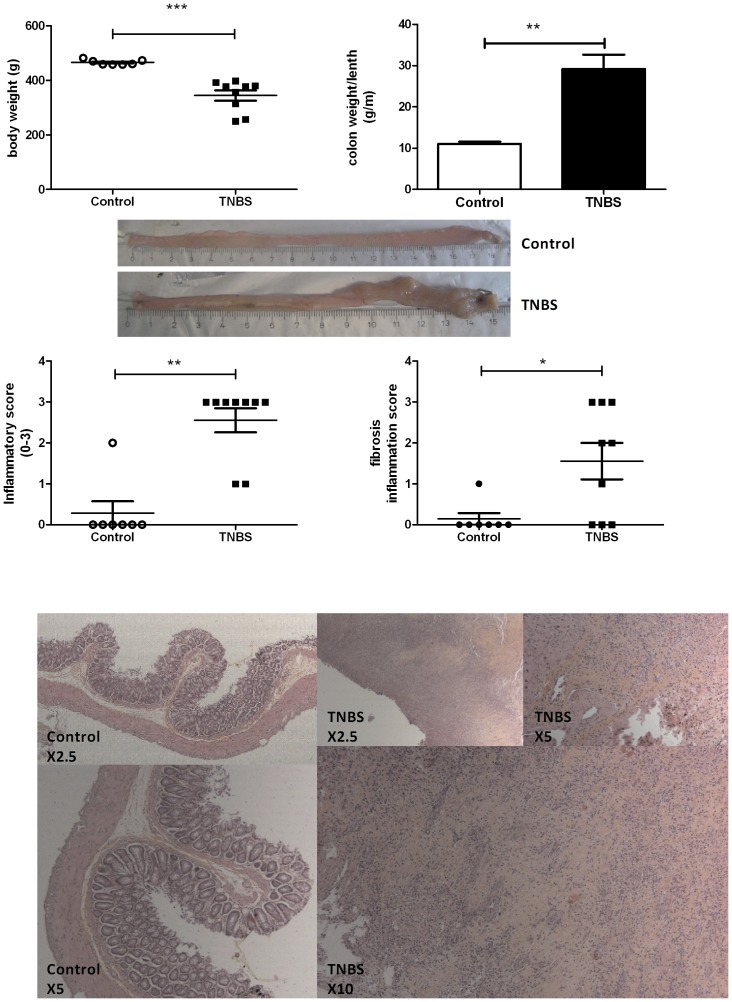
Validation of chronic TNBS-induced colitis. Chronic colitis was induced by weekly intrarectal injection of TNBS for 6 weeks. MRC was performed 1 week after the last injection. (A) Body weight on day of MRC. (B) Colon weight/length (g/m) in the control and TNBS group. (C) Colon macroscopy. (D) Histological score of inflammation from 0 (no inflammation) to 3 (severe inflammation). (E) Histological score of fibrosis from 0 (no fibrosis) to 3 (severe fibrosis). (F) Hematoxylin-eosin stained tissue of control and TNBS groups. In the TNBS sections, chronic inflammation and fibrosis occasioned architectural disorders, lymphocytic infiltrate and fibrin deposits. (Magnification, ×2.5, ×5, ×10). Values are means ± SEM.

**Figure 2:**
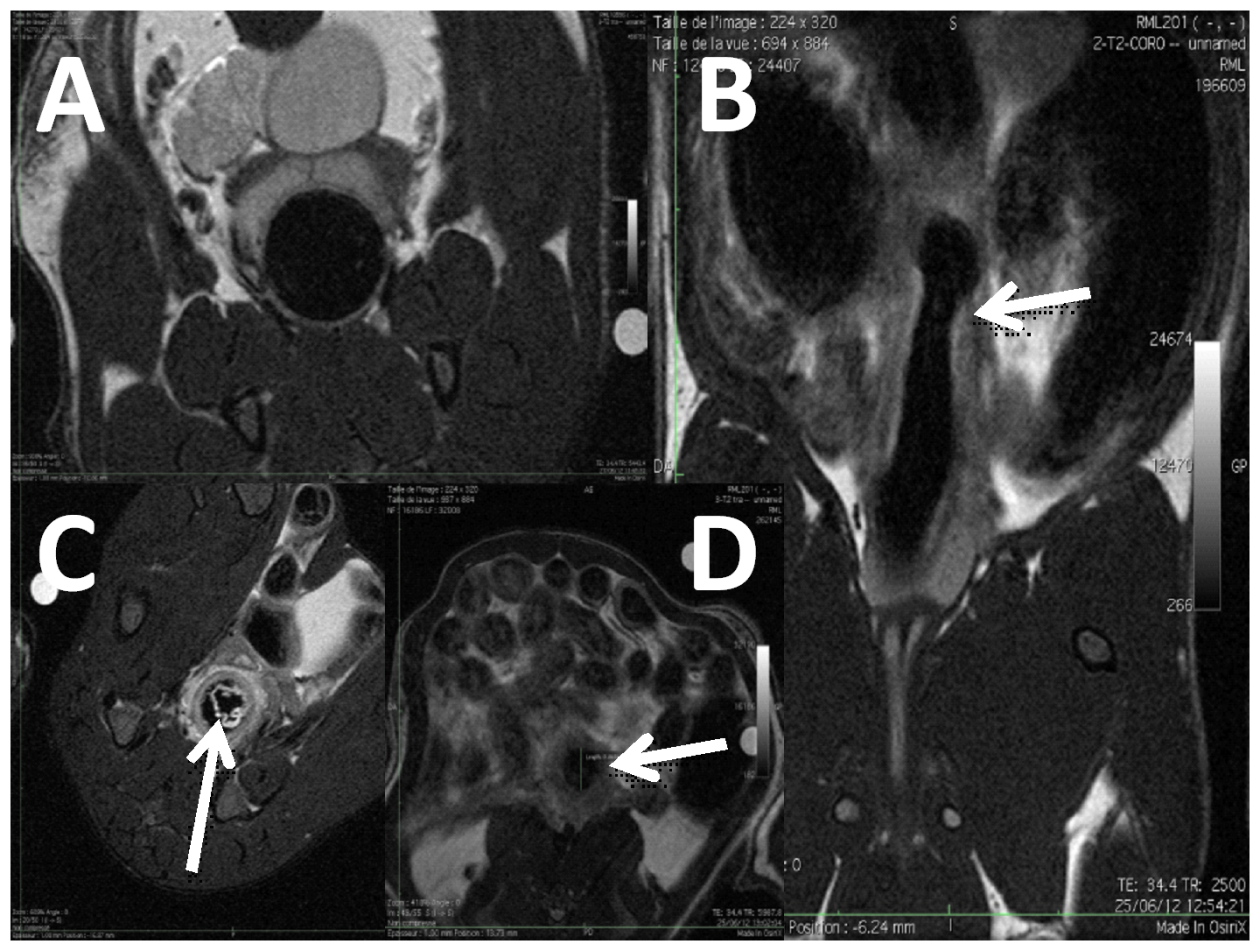
MRC of rats, T2w abdominal images. Chronic colitis was induced by weekly intrarectal injection of TNBS for 6 weeks. MRC was performed 1 week after the last injection. (A) Axial image of a control rat: colon wall is thin and regular. (B) Coronal image of a chronic TNBS rat: the arrow indicates stenosis with prior dilatation. (C) Axial image of a TNBS rat (T2w with fat suppression): the arrow indicates colon mucosal detachment. (D) Axial image of a TNBS rat: the arrow indicates colon wall thickening.

The rats recovered well after anesthesia and MRC. Image quality was considered as excellent in 5, good in 7 and medium in 4 examinations, with no difference between groups (p = 0.5338). Colon wall thickness was significantly higher in the TNBS than in the control group with maximal thickness at 2.38±0.63 mm versus 0.74±0.08 mm, p<0.0001, and minimal thickness at 1.33±0.34 mm versus 0.48±0.08 mm, p<0.0001. Both maximal and minimal thickness correlated with colon weight/length ratio (r = 0.9024, p<0.0001 and r = 0.8803, p<0.0001 respectively) (**Figure 1**). Wall signal intensity on T2w images was significantly higher in rats with TNBS (9040±2424 versus 6192±766, p = 0.01). Ratio percentage of luminal narrowing was significantly increased in the TNBS group (50.08±18.23% versus 10.33±9.01%, p = 0.0001). Presence of stenosis was observed in 7 out of 9 rats with TNBS and in none of the 7 controls (p = 0.005) (**Figure 2**). Mucosal detachment was found in 4 out of 9 rats with TNBS and in none of the 7 controls (p = 0.09). There was no difference between the two groups regarding presence of tubular aspect (p = 0.47) and wall signal intensity on T1 images (p = 0.46).

### Correlation of MRC parameters and fibrosis parameters

MRC measurement of wall thickness was significantly associated with fibrosis scores, and α-SMA production (r = 0.5813, p = 0.0182 for maximal thickness and r = 0.6382, p = 0.0078 for minimal thickness). **Table 1** summarizes correlation between wall thickness and fibrosis parameters.

**Table 1:** Association between MRC criteria and fibrosis parameters.

p (r)	Fibrosis score	TGF-β	IL-13	αSMA	P-Smad2/3
	*Spearman*	*Spearman*	*Pearson*	*Spearman*	*Spearman*
**Maximal thickness**	**0.0006**	0.7492	0.2028	**0.0182**	**0.0054**
	(0.7614)	(0.08683)	(0.3625)	(0.5813)	(0.6792)
**Minimal thickness**	**0.0119**	0.6993	0.1294	**0.0078**	**0.0412**
	(0.611)	(0.1059)	(0.4255)	(0.6382)	(0.5321)
**T2 wall signal**	**0.0384**	0.5276	0.226	0.1309	**0.0198**
	(0.5214)	(−0.1706)	(0.3457)	(0.3941)	(0.5929)
**Luminal narrowing**	0.0895	0.5276	0.0964	**0.0108**	0.0577
	(0.4383)	(0.1706)	(0.4618)	(0.56176)	(0.5)

P value in bold is significantly different (p<0.05) while p value in italics represents a trend (0.05<p<0.10).

Wall signal intensity on T2w images correlated with histological fibrosis score (Spearman r = 0.5214, p = 0.0384), and p-Smad2/3 (Spearman r = 0.5929, p = 0.0198). Wall signal intensity on T2w did not correlate with IL-13 (Pearson r = 0.3457, p = 0.2260), α-SMA (Spearman r = 0.3941, p = 0.1309), TGF-β (Spearman r = −0.1706, p = 0.5276), IGF-1 (Spearman r = 0.3719, p = 0.2483) or vimentin (Pearson r = 0.1149, p = 0.6834).

Luminal narrowing calculated by MRC correlated with α-SMA (Spearman r = 0.56176, p = 0.0108). Luminal narrowing tended to correlate with histological score of fibrosis (Spearman r = 0.4383, p = 0.0895), IL-13 (Pearson r = 0.4618, p = 0.0964), p-Smad2/3 (Spearman r = 0.5000, p = 0.0577) and vimentin (Pearson r = 0.5011, p = 0.0570). Luminal narrowing did not correlate with TGF-β (Spearman r = 0.1706, p = 0.5276) or IGF-1 (Spearman r = 0.2893, p = 0.2957).

**Figure 3:**
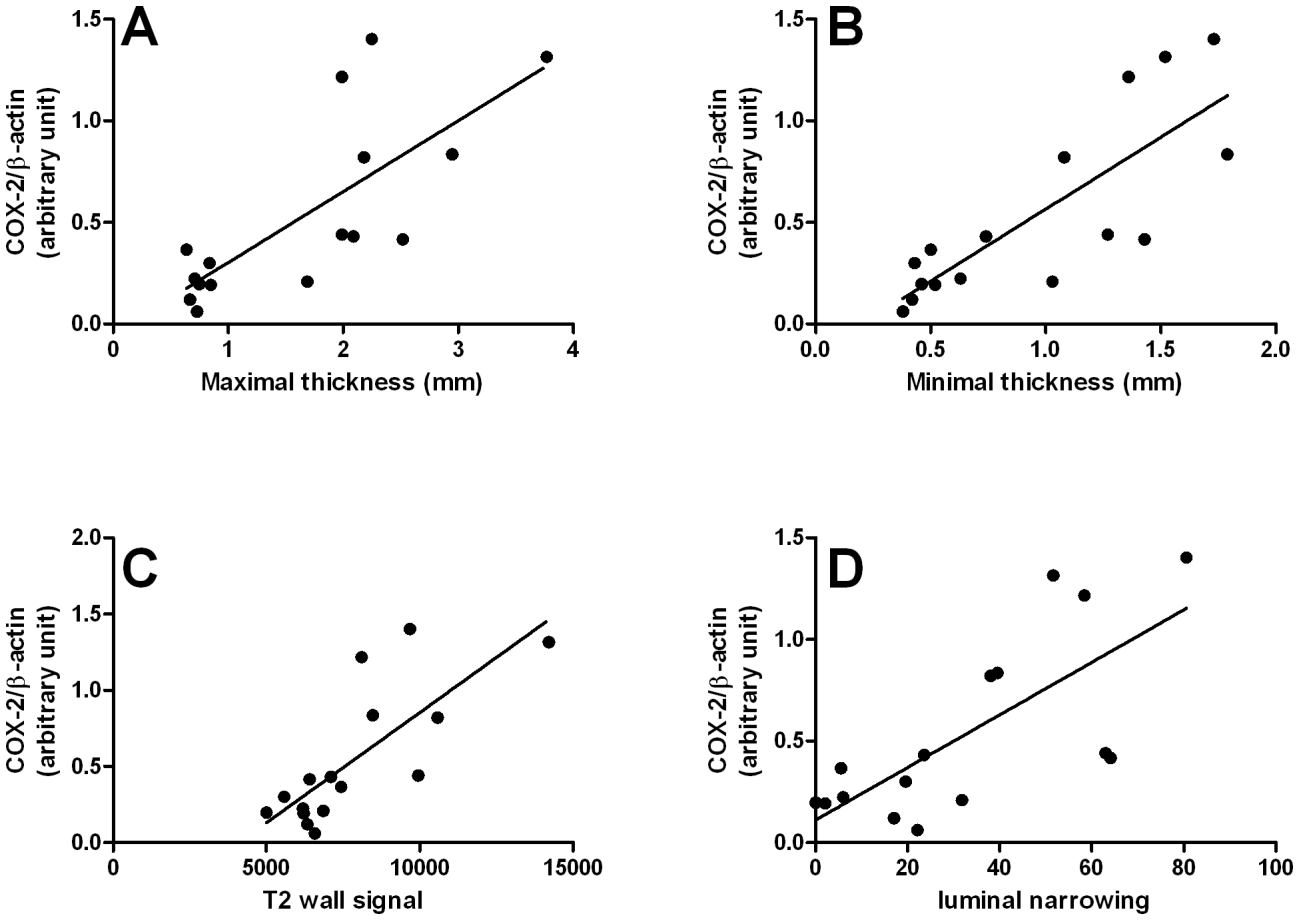
Association between MRC criteria and colon COX-2 expression in rats with chronic colitis. Chronic colitis was induced in rats by intrarectal injection of TNBS repeated on a weekly basis from day 0 to day 35. MRC was performed at day 42. Maximal and minimal thickness, colon wall signal intensity and luminal narrowing was measured on MR images. Colon COX-2 expression was measured by western blot. Simple linear regression between maximal (A°, minimal (B), colon wall signal intensity (C), luminal narrowing (D) and COX-2 expression. score. Pearson correlation was performed.

MRC parameters including, minimal and maximal thickness, wall signal intensity on T2w images and calculated luminal narrowing correlated with COX-2 but not with inflammatory markers like TNF-α and IL-1β (**Figure 3**, **Table 2**).

**Table 2:** Association between MRC criteria and inflammatory parameters.

p (r)	TNFα	IL1β	COX2
	*Spearman*	*Spearman*	*Pearson*
**Maximal thickness**	0.48	**0.01**	**0.0007**
	(−0.1884)	(0.4114)	(0.7564)
**Minimal thickness**	0.28	0.19	**0.0002**
	(−0.2824)	(0.3714)	(0.8052)
**T2 wall signal**	0.19	**0.0052**	**0.0005**
	(0.3412)	(0.7654)	(0.7654)
**Luminal narrowing**	0.93	0.23	**0.0014**
	(−0.02353)	(0.3407)	(0.7287)

P value in bold is significantly different (p<0.05).

## Discussion

The current study aimed to validate MRC as a tool for discriminating intestinal fibrosis in rats with chronic TNBS-induced colitis. Our results show a positive relationship between MRC criteria, histological score of fibrosis and fibrosis parameters.

MRC is able to discriminate rats with fibrosis and control rats. Similar to previous reports [11], we observed colonic fibrosis demonstrated by stiffened colon, histopathological fibrosis and elevation of fibrosis markers in rats with chronic TNBS colitis. In this study, we noted numerous pathological signs on MR including, wall thickening, significant luminal narrowing and mural high signal intensity in T2-weighted sequence. Our results are based on morphological changes in the colon wall and on analysis of signal changes. Others approaches have been developed including evaluation of magnetization to identify excessive collagen in fibrosis lesions. This technique has been used in brain imaging for more than a decade and is of major interest in management of multiple sclerosis [21]. Applied to the intestinal wall these experiments have allowed description of abnormal magnetization in the colon wall in animal models [18], [22]. However, morphological studies and analysis of intestinal wall signal in T2- and T1- weighted sequences remain the most common approach in MR studies. Moreover, presence of an irreversible stricture is more relevant than collagen content in the colon wall. Indeed, stenosis is one of the major landmarks of progressive irreversible damage of the intestinal wall. These stenoses are usually a combination of inflammatory and fibrosis elements. In clinical practice only lack of response to potent anti-inflammatory strategies (mainly steroids or anti-TNF) is able to identify strictures requiring surgery, especially in CD patients. In our report, MR was able to identify luminal narrowing, reflecting alteration of colonic diameter leading to stenosis. This MR aspect was associated with relevant biological landmarks of fibrosis (α-SMA) and tended to correlate with fibrosis score. Luminal narrowing was also obviously related to increase in mural thickness in our observations, see figure 2.

Increased mural thickness has been described in mice with chronic Dextran Sodium sulfate (DSS) [19]. In this study by the Leuven group, the authors differentiated mucosal thickening from more profound layer changes [19]. Mucosal thickness seemed to follow inflammation through repeated exposure to DSS as thickness of the *muscularis propria* seemed to follow progressive increase in the collagen content of the colonic wall. This suggests that measurement of thickening in the colon wall combined inflammatory and fibrosis contents. In addition to wall thickening, the colon wall signal was also modified and consisted in significant hyperintensity in T2-weighted sequence. We assessed this colon wall signal by measuring signal intensity in selected regions of interest (ROI), as widely validated in acute rodent colitis [16], [23], [24]. Others have described this hyperintensity using T2 mapping and observed a gradual regression of T2 values with increasing cycles of DSS. These results suggest a shift towards high hyperintensity in T2-weighted sequence in acute phase and towards lower hyperintensity in T2-weighted sequence in more chronic lesions. This is in accordance with our previous observation of high relationship between hyperintensity in T2-weighted sequence and inflammation in acute colitis [16]. Moreover, in our present report we also observed a numerically significant correlation between hyperintensity in T2-weighted sequence and remaining histological inflammation.

Persistence of inflammation is a theoretical limitation of animal model of intestinal fibrosis. This persistence is observed in chronic TNBS [11] as in our work, in peptidoglycan-polysaccharide infected mice [22] and in chronic DSS colitis. Indeed, Breynaert *et al.* found that repeated exposure to DSS can induce persistent inflammation in parallel with fibrosis lesions [19]. Consequently assessing the respective part of each phenomenon is a challenging condition. We speculated that this inflammatory persistence resulted from the experimental design used in most studies. In our work, rats underwent MRI 7 to 10 days after the last intra-rectal administration of TNBS. TNBS administration was scheduled at regular time points but with increasing dosage that may have led to up regulation of inflammatory markers. A longer time period between the last TNBS injection and MRC examination could result in less pronounced inflammation. Recently, Breynaert *et al.* reported that prolonged recovery after 2 cycles of DSS in mice with chronic DSS-induced colitis was associated with decreased inflammation and progressive accumulation of collagen in the colon wall [19].

We did not observe any change in intensity of colon wall in T1 sequences. This probably reflects absence of hemorrhagic lesion and severe acute inflammation at time of MRC evaluation. This result contrasts with the association of hyper T1 signal in acute inflammation [16].

To summarize recent contributions to our knowledge of MR changes in the colon wall, it could be speculated that it is a continuous process from acute lesions with hyperintensity in T1- and T2-weighted sequence, followed by progressive decrease in signal while collagen content increases over time. In the TNBS model this process seems to be able to mimic CD lesion with luminal narrowing. To confirm this hypothesis repeated MR examination and longitudinal studies are mandatory.

Among biological parameters, if some are obviously related to acute phase of inflammation (IL-1β, TNFα) others are related to chronic fibrosis lesion (collagen, α-SMA) [19]. However, this is a double-edged sword which does not give the whole picture. Breynaert *et al*. also described a colonic gene profile associated with both inflammation and fibrosis. COX-2 is a marker which for decades has clearly been associated with acute inflammation [25], [26], [27], [28] but which also increases significantly with time in fibrosis models, pulmonary fibrosis [29] and colonic fibrosis included [30]. In our observation a similar relationship between COX-2 and MR parameters linked both fibrosis and inflammation. This observation of common markers in the two phases pleads for a progressive switch from inflammation to fibrosis. Similarly, infection of mice with Salmonella led to enterocolitis with mucosal and submucosal inflammation as well as with fibrosis as shown by an impressive time dependent collagen deposition with a parallel increase of COX-2 [30].

In conclusion, our report demonstrates the feasibility of MRC in rodent models of chronic colitis. It establishes a link between changes in MR in the colonic wall and fibrosis. In addition, our report highlights the challenge of distinguishing fibrosis from persisting inflammation in a lesion. Repeated MR evaluation may help to better understand intestinal damage in IBD patients and may be the key to better understanding of this challenging condition.

## References

[1] Pariente B, Cosnes J, Danese S, Sandborn WJ, Lewin M, et al (2011) Development of the Crohn's disease digestive damage score, the Lemann score. Inflamm Bowel Dis 17: 1415–1422. 21560202 10.1002/ibd.21506 PMC3116198

[2] Cosnes J (2013) Measuring structural damage in Crohn's disease. Gastroenterol Hepatol (N Y) 9: 103–104. 23983655 PMC3754768

[3] Cosnes J, Gower-Rousseau C, Seksik P, Cortot A (2011) Epidemiology and natural history of inflammatory bowel diseases. Gastroenterology 140: 1785–1794. 21530745 10.1053/j.gastro.2011.01.055

[4] Peyrin-Biroulet L, Harmsen WS, Tremaine WJ, Zinsmeister AR, Sandborn WJ, et al (2012) Surgery in a population-based cohort of Crohn's disease from Olmsted County, Minnesota (1970–2004). Am J Gastroenterol 107: 1693–1701. 22945286 10.1038/ajg.2012.298 PMC3572861

[5] Burke JP, Mulsow JJ, O'Keane C, Docherty NG, Watson RW, et al (2007) Fibrogenesis in Crohn's disease. Am J Gastroenterol 102: 439–448. 17156147 10.1111/j.1572-0241.2006.01010.x

[6] Cosnes J, Nion-Larmurier I, Beaugerie L, Afchain P, Tiret E, et al (2005) Impact of the increasing use of immunosuppressants in Crohn's disease on the need for intestinal surgery. Gut 54: 237–241. 15647188 10.1136/gut.2004.045294 PMC1774826

[7] Rieder F, Fiocchi C (2009) Intestinal fibrosis in IBD–a dynamic, multifactorial process. Nat Rev Gastroenterol Hepatol 6: 228–235. 19347014 10.1038/nrgastro.2009.31

[8] Fiocchi C, Lund PK (2011) Themes in fibrosis and gastrointestinal inflammation. Am J Physiol Gastrointest Liver Physiol 300: G677–683. 21415411 10.1152/ajpgi.00104.2011 PMC3094134

[9] Rieder F, Kessler SP, West GA, Bhilocha S, de la Motte C, et al (2011) Inflammation-induced endothelial-to-mesenchymal transition: a novel mechanism of intestinal fibrosis. Am J Pathol 179: 2660–2673. 21945322 10.1016/j.ajpath.2011.07.042 PMC3204019

[10] Morris GP, Beck PL, Herridge MS, Depew WT, Szewczuk MR, et al (1989) Hapten-induced model of chronic inflammation and ulceration in the rat colon. Gastroenterology 96: 795–803. 2914642

[11] Stidham RW, Xu J, Johnson LA, Kim K, Moons DS, et al (2011) Ultrasound elasticity imaging for detecting intestinal fibrosis and inflammation in rats and humans with Crohn's disease. Gastroenterology 141: 819–826 e811. 21784048 10.1053/j.gastro.2011.07.027 PMC4934420

[12] Rimola J, Rodriguez S, Garcia-Bosch O, Ordas I, Ayala E, et al (2009) Magnetic resonance for assessment of disease activity and severity in ileocolonic Crohn's disease. Gut 58: 1113–1120. 19136510 10.1136/gut.2008.167957

[13] Panes J, Bouzas R, Chaparro M, Garcia-Sanchez V, Gisbert JP, et al (2011) Systematic review: the use of ultrasonography, computed tomography and magnetic resonance imaging for the diagnosis, assessment of activity and abdominal complications of Crohn's disease. Aliment Pharmacol Ther 34: 125–145. 21615440 10.1111/j.1365-2036.2011.04710.x

[14] Ordas I, Rimola J, Ripolles T, García-Bosch O, Rodríguez S, et al (2011) Accuracy of MRI to Assess Therapeutic Responses and Mucosal Healing in Crohn's Disease. Gastroenterology 140: S–73.

[15] Rimola J, Ordás I, Rodriguez S, García-Bosch O, Aceituno M, et al (2011) Magnetic resonance imaging for evaluation of Crohn's disease: Validation of parameters of severity and quantitative index of activity. Inflammatory Bowel Diseases 17: 1759–1768. 21744431 10.1002/ibd.21551

[16] Charpentier C, Marion-Letellier R, Savoye G, Nicol L, Mulder P, et al (2012) Magnetic resonance colonography in rats with TNBS-induced colitis: a feasibility and validation study. Inflamm Bowel Dis 18: 1940–1949. 22262626 10.1002/ibd.22897

[17] Savoye-Collet C, Savoye G, Koning E, Dacher JN, Lerebours E (2011) Fistulizing perianal Crohn's disease: contrast-enhanced magnetic resonance imaging assessment at 1 year on maintenance anti-TNF-alpha therapy. Inflamm Bowel Dis 17: 1751–1758. 21744430 10.1002/ibd.21568

[18] Adler J, Rahal K, Swanson SD, Schmiedlin-Ren P, Rittershaus AC, et al (2013) Anti-tumor necrosis factor alpha prevents bowel fibrosis assessed by messenger RNA, histology, and magnetization transfer MRI in rats with Crohn's disease. Inflamm Bowel Dis 19: 683–690. 23429466 10.1097/MIB.0b013e3182802c32

[19] Breynaert C, Dresselaers T, Perrier C, Arijs I, Cremer J, et al (2013) Unique gene expression and MR T2 relaxometry patterns define chronic murine dextran sodium sulphate colitis as a model for connective tissue changes in human Crohn's disease. PLoS One 8: e68876. 23894361 10.1371/journal.pone.0068876 PMC3720888

[20] Mustafi D, Fan X, Dougherty U, Bissonnette M, Karczmar GS, et al (2010) High-resolution magnetic resonance colonography and dynamic contrast-enhanced magnetic resonance imaging in a murine model of colitis. Magn Reson Med 63: 922–929. 20373393 10.1002/mrm.22229 PMC3086519

[21] Richert ND, Frank JA (1999) Magnetization transfer imaging to monitor clinical trials in multiple sclerosis. Neurology 53: S29–32. 10496208

[22] Adler J, Swanson SD, Schmiedlin-Ren P, Higgins PD, Golembeski CP, et al (2011) Magnetization transfer helps detect intestinal fibrosis in an animal model of Crohn disease. Radiology 259: 127–135. 21324841 10.1148/radiol.10091648 PMC3064818

[23] Kohnke T, Gomolka B, Bilal S, Zhou X, Sun Y, et al (2013) Acetylsalicylic Acid reduces the severity of dextran sodium sulfate-induced colitis and increases the formation of anti-inflammatory lipid mediators. Biomed Res Int 2013: 748160. 24083240 10.1155/2013/748160 PMC3780524

[24] Michael S, Keubler LM, Smoczek A, Meier M, Gunzer F, et al (2013) Quantitative phenotyping of inflammatory bowel disease in the IL-10-deficient mouse by use of noninvasive magnetic resonance imaging. Inflamm Bowel Dis 19: 185–193. 22570250 10.1002/ibd.23006

[25] Mbodji K, Charpentier C, Guerin C, Querec C, Bole-Feysot C, et al (2012) Adjunct therapy of n-3 fatty acids to 5-ASA ameliorates inflammatory score and decreases NF-kappaB in rats with TNBS-induced colitis. J Nutr Biochem 24: 700–705. 22841543 10.1016/j.jnutbio.2012.03.022

[26] Hassan A, Ibrahim A, Mbodji K, Coeffier M, Ziegler F, et al (2010) An alpha-linolenic acid-rich formula reduces oxidative stress and inflammation by regulating NF-kappaB in rats with TNBS-induced colitis. J Nutr 140: 1714–1721. 20724486 10.3945/jn.109.119768

[27] Ibrahim A, Mbodji K, Hassan A, Aziz M, Boukhettala N, et al (2011) Anti-inflammatory and anti-angiogenic effect of long chain n-3 polyunsaturated fatty acids in intestinal microvascular endothelium. Clin Nutr 30: 678–687. 21632157 10.1016/j.clnu.2011.05.002

[28] Fukata M, Chen A, Klepper A, Krishnareddy S, Vamadevan AS, et al (2006) Cox-2 is regulated by Toll-like receptor-4 (TLR4) signaling: Role in proliferation and apoptosis in the intestine. Gastroenterology 131: 862–877. 16952555 10.1053/j.gastro.2006.06.017 PMC2169292

[29] Liu F, Mih JD, Shea BS, Kho AT, Sharif AS, et al (2010) Feedback amplification of fibrosis through matrix stiffening and COX-2 suppression. J Cell Biol 190: 693–706. 20733059 10.1083/jcb.201004082 PMC2928007

[30] Mansson LE, Montero M, Zarepour M, Bergstrom KS, Ma C, et al (2012) MyD88 signaling promotes both mucosal homeostatic and fibrotic responses during Salmonella-induced colitis. Am J Physiol Gastrointest Liver Physiol 303: G311–323. 22679002 10.1152/ajpgi.00038.2012

